# Evolution of Resistant Mutants in *Pseudomonas aeruginosa* Persister Cells Under Meropenem Treatment

**DOI:** 10.3390/microorganisms13071672

**Published:** 2025-07-16

**Authors:** Jie Feng, Yifan Bian, Congjuan Xu, Zhihui Cheng, Yongxin Jin, Shouguang Jin, Weihui Wu

**Affiliations:** State Key Laboratory of Medicinal Chemical Biology, Key Laboratory of Molecular Microbiology and Technology of the Ministry of Education, Department of Microbiology, College of Life Sciences, Nankai University, Tianjin 300071, China; 1120220628@mail.nankai.edu.cn (J.F.); 2120231464@nankai.edu.cn (Y.B.); xucongjuan96@163.com (C.X.); zhihuicheng@nankai.edu.cn (Z.C.); yxjin@nankai.edu.cn (Y.J.); nksjin@nankai.edu.cn (S.J.)

**Keywords:** *Pseudomonas aeruginosa*, persister cells, antibiotic resistance, meropenem, evolutionary pathway, *oprD*, *mexR*

## Abstract

Bacterial persisters are dormant cells that survive antibiotic treatment, serving as a reservoir for the emergence of resistant mutations. The evolution of antibiotic resistance poses a significant challenge to public health. In this study, we investigated the development of resistance in *Pseudomonas aeruginosa* persister cells by exposing the reference strain PA14 to meropenem and tracked the emergence of resistance mutations over serial passages. Whole-genome sequencing of the populations or individual resistant strains revealed evolutionary trajectories. In the initial passages, low-level meropenem-resistant mutants harbored various mutations, accompanied by increasing population survival. Then, mutations in the *oprD* gene appeared, followed by mutation in the *mexR* gene in most of the cells, leading to high-level meropenem resistance and collateral resistance to ciprofloxacin. Our study provides insights into the evolutionary pathways of *P. aeruginosa* under lethal antibiotic pressure, highlighting the dynamic interplay between persister cells and the emergence of resistance mutations.

## 1. Introduction

Antibiotic resistance has emerged as one of the most pressing global public health challenges. A large proportion of resistant infections are caused by a group of bacterial pathogens including *Enterococcus faecium*, *Staphylococcus aureus*, *Klebsiella pneumoniae*, *Acinetobacter baumannii*, *Pseudomonas aeruginosa*, and *Enterobacter* spp., which are called ESKAPE [1]. These organisms possess a range of intrinsic and acquired resistance mechanisms that enable them to escape the effects of antimicrobial agents. The persistent use of antibiotics has driven the emergence of multidrug-resistant (MDR) and extensively drug-resistant (XDR) strains, rendering many conventional antibiotics ineffective [2]. ESKAPE pathogens are associated with high mortality rates and significantly increased healthcare costs. Among ESKAPE, *P. aeruginosa* is an opportunistic human pathogen that can cause life-threatening acute and chronic infections [3]. Carbapenems have been considered the most active and potent agents against MDR Gram-negative pathogens [4]. However, efflux pump upregulation, porin mutations, and β-lactamase production, etc., increase bacterial resistance to carbapenems [5,6,7]. Carbapenem-resistant *P. aeruginosa* (CRPA) is disseminated globally and constitutes a significant threat to public health [8,9]. It is in the “critical” category of the World Health Organization (WHO)’s priority list of bacterial pathogens, for which research and development of new antibiotics is urgently required [10].

*P. aeruginosa* is a leading cause of nosocomial infections, with carbapenem resistance posing a critical therapeutic challenge [8,9]. Central to this resistance are two chromosomal mechanisms: loss of the OprD porin and hyperactivation of RND efflux pumps via *mexR* mutations. The OprD porin facilitates carbapenem uptake, and its inactivation—through frameshift mutations, premature stop codons, or large-fragment deletions—reduces membrane permeability, elevating imipenem or meropenum MICs by 4–32-fold. Notably, 79.1% of carbapenem-resistant clinical isolates show *oprD* downregulation, while 20.9% exhibit overexpression of efflux pumps or carbapenemases [11]. Concurrently, *mexR* mutations derepress the MexAB-OprM efflux pump, a major transporter of diverse antibiotics, including β-lactams, fluoroquinolones, aminoglycosides, etc. Structural studies reveal that *mexR* mutations or oxidative-stress-induced disulfide bond formation trigger conformational changes in MexR’s DNA-binding domain, abolishing its repressor function and leading to *mexAB*-*oprM* over expression [12]. It has been found that *mexR* mutants play major roles in antibiotic evolution and drive collateral resistance. Clinically, synergistic *oprD* loss and MexAB-OprM hyperactivation create a “dual-hit” resistance mechanism, observed in 89% of carbapenem-resistant strains [13].

Bacterial evolution, driven by mechanisms such as mutation and horizontal gene transfer, is a key factor in the development of antimicrobial resistance [14,15,16]. Bacterial persisters are a subpopulation of dormant cells that survive lethal antibiotic treatment through mechanisms such as toxin–antitoxin (TA) systems and metabolic dormancy, leading to chronic and recurrent infections [17,18,19,20]. *P. aeruginosa* utilizes toxin–antitoxin (TA) systems as critical regulators for stress adaptation, persistence formation, and virulence expression. TA systems typically comprise a stable toxin that disrupts essential cellular processes and a labile antitoxin that neutralizes toxicity. Recent genomic analyses reveal that *P. aeruginosa* harbors 12–15 type II TA systems across major strains such as PA14 (13 systems), PAO1 (12 systems), and ATCC 27853 (15 systems), with significant variation in mobile genetic elements [21,22,23]. TA systems further drive metabolic reprogramming for persistence. The PA14_51010 proteins in the PA14 strain reduce intracellular NAD+ levels to induce metabolic dormancy and antibiotic tolerance, while the ParE toxin inhibit DNA gyrase to halt replication under nutrient stress. This persistence phenotype facilitates chronic infections, as seen in cystic fibrosis lungs where TA systems coordinate long-term colonization via (p)ppGpp signaling networks [24,25]. In addition, persister cells may serve as a reservoir for the development of resistance mutations over time, as their survival under antibiotic pressure provides an opportunity for mutations to arise [26,27]. Understanding the interplay between persister formation and the emergence of resistance mutations is crucial for developing novel therapeutic strategies to combat persistent infections and prevent the evolution and spread of antibiotic resistance.

In studies of bacterial resistance evolution, in vitro serial passaging experiments have been widely used, in which bacteria are grown in increasing concentrations of antibiotics to select resistant mutants [28,29]. However, resistant mutants arising from non-growing persister are likely to be neglected in the assay. Our hypothesis was that the distinctive physiological status and gene expression profile of persisters might affect the evolution trajectory of resistance. Thus, in this study, we examined the resistance development of *P. aeruginosa* persister cells following treatment with a lethal dosage of meropenem and characterized the mutation sites as well as collateral resistance.

## 2. Materials and Methods

### 2.1. Bacterial Strains, Plasmids, and Primers

The bacterial strains, plasmids, and primers used in this study are detailed in Supplementary Table S1. *P. aeruginosa* PA14 (ATCC 27853) served as the primary wild-type strain. All strains were cultured aerobically in Luria–Bertani (LB) medium (1% tryptone, 0.5% yeast extract, 1% NaCl, pH 7.4 ± 0.2) at 37 °C with constant shaking at 200 rpm. Long-term storage utilized glycerol stocks (25% *v*/*v*) prepared from mid-log-phase cultures (OD_600_ = 0.8–1.0) and stored at −80 °C, with working strains subcultured on LB agar plates for no more than two passages to minimize genetic drift.

### 2.2. Antimicrobial Susceptibility Test

Minimum inhibitory concentrations (MICs) of meropenem, ciprofloxacin, and tobramycin were determined by the broth microdilution method in cation-adjusted Mueller–Hinton broth (CAMHB, Oxoid CM0405) following Clinical and Laboratory Standards Institute (CLSI) M100-Ed34 (2024) guidelines [30]. Mid-log-phase cultures were adjusted to 0.5 McFarland standard (~1 × 10^8^ CFU/mL) in sterile saline using a Densimat densitometer (BioMérieux, Marcy-l’Étoile, France) and then diluted 1:100 in CAMHB to achieve a final inoculum of 5 × 10^5^ CFU/well in 96-well microtiter plates. Two-fold serial dilutions of antibiotics (meropenem: 0.015–256 μg/mL; ciprofloxacin: 0.008–32 μg/mL; tobramycin: 0.25–128 μg/mL) were prepared using an electronic multichannel pipette (Eppendorf (Hamburg, Germany) EpMotion 5075t) to ensure volumetric accuracy. Each plate included *P. aeruginosa* ATCC 27853 as quality control strains, with the MIC defined as the lowest concentration showing ≥90% growth inhibition after 18 ± 2 h of incubation at 35 ± 2 °C under visual inspection.

### 2.3. Bacteria Killing Assay

Briefly, overnight bacterial cultures were diluted into fresh LB to an OD_600_ of 0.05. The bacteria were cultured for 2 h until the OD_600_ reached approximately 0.8~1.0. Then, the bacteria were treated with 8 μg/mL meropenem. At each indicated time point, an aliquot of the cultures was taken out. The bacteria were washed with 0.9% NaCl three times, followed by serial dilution and plating on LB agar for colony-forming unit (CFU) counting [31,32]. All experiments were conducted in biological triplicates.

### 2.4. Experimental Evolution of Persister Cells

The overnight culture of wild-type PA14 was diluted 1:100 in 3 mL of fresh LB. The bacteria were grown at 37 °C, 200 rpm until the OD_600_ reached 0.8–1.0, followed by treatment with 8 μg/mL meropenem for 6 h. A total of 100 μL of the bacteria was used to determine the number of live bacteria by plating. The remaining cells were subjected to centrifugation in 0.3 M sucrose at 12,000 rpm to remove dead cell debris. The bacteria were then resuspended in 3 mL of fresh LB and culture to an OD_600_ of 1.0. A total of 100 μL of the bacteria was plated on plates containing indicated antibiotics (meropenem, ciprofloxacin, and tobramycin) at different concentrations to determine resistance mutation rates. The remaining bacteria were subjected to the next round of meropenem (8 μg/mL) treatment. This process was repeated until 8 μg/mL of meropenem was no longer capable of killing the bacteria. For the control group, cultures were grown under the same conditions (37 °C, 200 rpm) until the OD_600_ reached 0.8–1.0. Similarly, 100 μL of the bacteria was plated on plates containing the indicated antibiotics at different concentrations. The remaining bacteria were processed in accordance with the experimental group and subjected to the same number of rounds as the experimental group.

### 2.5. Genomic Sequencing

Individual clones were subjected to next-generation whole-genome sequencing conducted by Shanghai Honsun Biological Technology Company. Whole-genome sequencing with a depth of 200× of the heterogenous bacterial pools was conducted by the Tianjin Uniteomics Company (Tianjin, China). Briefly, bacterial genomic DNA was extracted with a DNA purification kit (Tiangen Biotech, Beijing, China). Fragments smaller than 500 bp were obtained from 200 ng genomic DNA by sonication (Covaris S220, Covaris, Woburn, MA, USA), followed by end treatment and adaptor ligation. Adaptor-ligated DNA fragments of about 470 bp were recovered and then PCR-amplified for six cycles, and the PCR products were cleaned up, validated using a Qsep100 (Bioptic, Taiwan, China), and quantified by a Qubit3.0 Fluorometer (Invitrogen, Carlsbad, CA, USA). Sequencing was carried out using a 2 × 150 paired-end (PE) configuration on an Illumina Hiseq instrument according to manufacturer’s instructions (Illumina, San Diego, CA, USA). The data were aligned to the PA14 reference genome (NC_002516.2) via the BW2 software (version 0.7.12). Single-nucleotide variation (SNV) or InDel mutation were detected using the software Samtools (version 1.1) and the Unified Genotyper module from GATK (version 4.5.0.0).

## 3. Results

**Figure 1:**
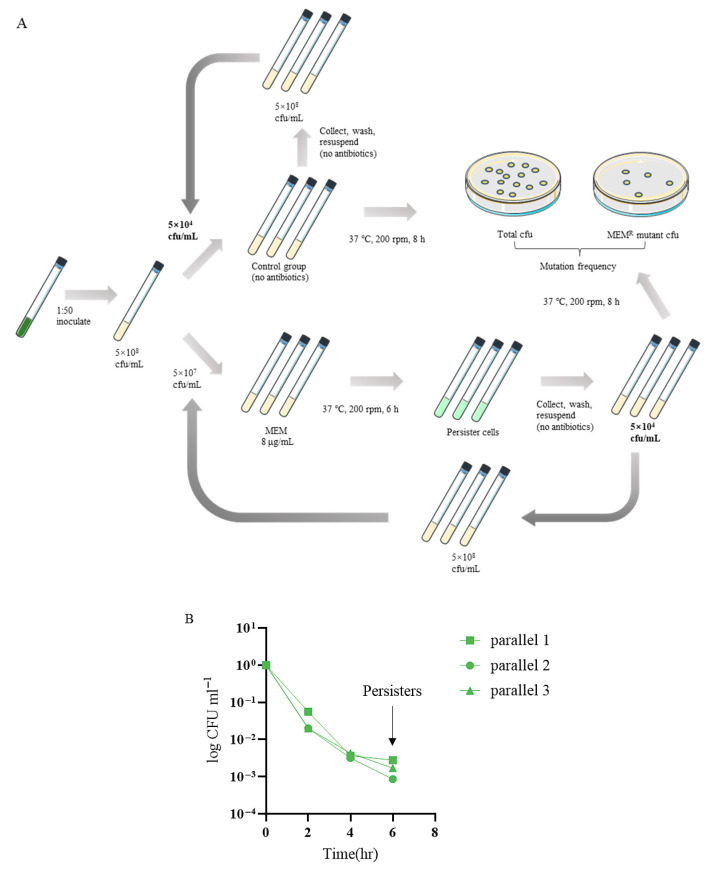
(**A**) The experimental flow chart. (**B**) The typical biphasic killing curve.

To investigate the dynamics and evolutionary trajectories of resistance developed from persister cells, we serially passaged the *P. aeruginosa* reference strain PA14 in a meropenem killing assay (Figure 1A). Three parallels of the bacteria were treated with 8 μg/mL meropenem (clinical break point), resulting in a typical biphasic killing curve (Figure 1B). At 6 h following the treatment, the bacteria were killed at a slow rate, representing a persister population (Figure 1B). The live bacteria were collected and grown in a fresh medium to an OD_600_ of 1.0, which was subjected to next round of meropenem killing. The dynamics of bacterial survival and antibiotic resistance frequencies were determined.

**Figure 2:**
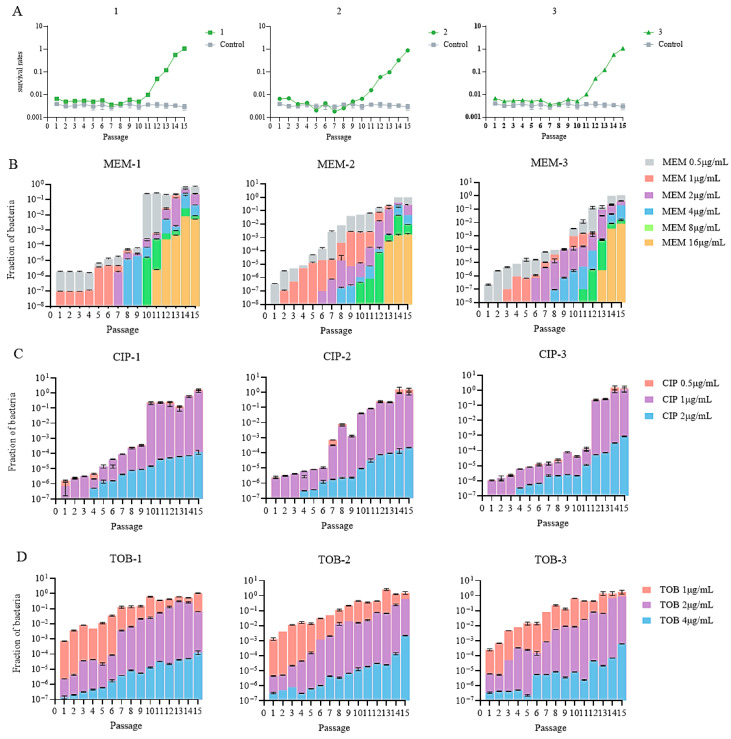
(**A**) Survival rates in the three parallels. (**B**–**D**) Mutation rates of the bacterial population after meropenem treatment against meropenem (MEM), ciprofloxacin (CIP), and tobramycin (TOB).

In the first 9–10 passages, the survival rates of the meropenem-treated groups remained at around 0.3%, followed by a quick increase to 100% over the next 5–6 passages (Figure 2A). In contrast, no increase in survival was observed in the cells passaged in antibiotic-free medium (Figure 2A). To determine the development of meropenem and collateral resistance after each passage, the recovered bacteria were plated on plates in the absence or presence of increasing amounts of meropenem, ciprofloxacin, and tobramycin. Clinically defined meropenem-resistant bacteria (MIC ≥ 16 μg/mL) arose at the 11th or 13th passage, in which the corresponding survival rates were 1% or 10% (Figure 2A,B). By 15 passages, around 1% of the bacteria were able to form colonies on plates containing 16 μg/mL meropenem (Figure 2B). Meanwhile, at least 0.01% of the bacteria were able to form colonies on plates containing ciprofloxacin and tobramycin at the concentrations of the clinical breakpoints (2 and 4 μg/mL, respectively) (Figure 2C,D).

To identify mutations associated with the development of resistance in these populations, the heterogenous bacterial pools in the final passage were subjected to whole-genome sequencing with a depth of 200×. Variations were identified at a 5% cut-off. A *mexR* T130P mutation was identified in all the three parallel populations with percentages of 100%, 99.55%, and 100%. Additionally, the proportions of *oprD* mutations in the populations were 41.36%, 69.35%, and 40.97%, respectively. We then isolated three single colonies on the 16 μg/mL meropenem plates from each of the parallels. The MICs of meropenem for the isolates reached 64 μg/mL. Meanwhile, the MICs of ciprofloxacin and tobramycin were both 0.5 μg/mL (Table 1).

Whole-genome sequencing revealed that all the isolates harbored the *mexR* T130P mutation with various *oprD* mutations (Table 2).

To validate the contribution of *mexR* and *oprD* gene mutations in antibiotic resistance, we constructed gene deletion mutants, the *mexR* T130P mutant and complementation strains, followed by antibiotic resistance evaluation (MIC test). Deletion of *mexR* in wild-type PA14 increased the MIC of meropenem and ciprofloxacin to 4 and 0.5 μg/mL, respectively, whereas the MIC of tobramycin was not affected. Deletion of *oprD* increased the MIC of meropenem to 8 μg/mL without affecting the MICs of ciprofloxacin and tobramycin. Meanwhile, deletion of both *oprD* and *mexR* increased the MIC of meropenem to 64 μg/mL, whereas the MICs of ciprofloxacin and tobramycin remained the same as the Δ*mexR* mutation (0.5 μg/mL) (Table 3). All the increased MICs were restored by complementation with a *mexR* gene (Table 3). These results verified that mutation of *oprD* increases bacterial resistance to meropenem, and mutation of *mexR* contributes to resistance to meropenem, ciprofloxacin, and tobramycin. In addition, mutations of *oprD* and *mexR* synergistically increase bacterial resistance to meropenem.

To explore the evolutionary trajectory, we isolated three single colonies on the 16 μg/mL meropenem plates at the earliest passage with the appearance of colonies from each of the parallels, i.e., passages 11th, 13th, and 13th passage of parallels 1, 2, and 3, respectively (Figure 2B). Whole-genome sequencing revealed mutations in *mexR* and *oprD* in all the strains. No mutation in other known resistance-related genes were identified. We then focused on the mutation trajectory of these two genes.

Additionally, we observed that, relative to the reference genome, the *lasR* gene in the control group contained two mutations, whereas *lasR* in the experimental groups remained consistent with the wild type.

From each parallel, we isolated six colonies on the plates containing 1, 2, 4, and 8 μg/mL meropenem that appeared at the earliest passages (Table 4). The MICs of the strains were determined (Table 4), and the *oprD* and *mexR* genes were sequenced (Table 5).

In all the strains isolated from 8 μg/mL meropenem plates, both the *mexR* T130P mutation and mutations in the *oprD* gene were present (Table 5). In the strains isolated from 4 μg/mL meropenem plates, fourteen strains contained mutations in *oprD*; however, no *mexR* mutation was identified (Table 5). In all the strains isolated from 2, 1, and 0.5 μg/mL meropenem plates, no mutation in either *mexR* or *oprD* was present. To identify mutations that rose before the *oprD* and *mexR* mutations, the bacterial population of each parallel at the sixth or seventh passage (the earliest passages with colonies on 2 μg/mL meropenem plates) were subjected to whole-genome sequencing with a depth of 200×.

Mutations in the genes *vgrG1b*, *phzD2*, *vgrG14*, and *cdrA* were present with percentage of 5.86–100% in all the sequenced populations from the 7th or 6th to the 15th passages (Tables S3–S5). Mutations in 28 genes were detected at a percentage of 5.59–26.73% at the seventh or sixth passages. However, those mutations were not detected in the clones from the 10–15th passages as well as single colonies and the whole population at the 15th passage (Tables S3–S5). Among those 28 mutated genes, *sltB1* (allele frequency 5.42%) in Group 1 (Generation 7th) encodes a lytic transglycosylase B that cuts peptidoglycan glycans.

## 4. Discussion

Bacterial persisters are cells that survived lethal dosages of antibiotics, which contribute to recurrent and chronic infections [33,34]. Meanwhile, these cells provide a reservoir for mutations that contribute to the development of antibiotic resistance [35,36]. The alarmone molecule (p)ppGpp and TA systems play important roles in persister formation [37]. They also affect global gene expression [38]. In addition, how (p)ppGpp, TA systems together with lethal antibiotic pressure affect mutation frequency and evolutionary trajectories remains to be explored. Here, in this study, we determined the resistance evolution and mutations by employing continuous passaging of *P. aeruginosa* cells under lethal concentrations of meropenem (16× MIC). Traditional investigations into bacterial resistance evolution overwhelmingly utilize sub-inhibitory concentrations (sub-MIC) of antibiotics to select for resistant mutants over serial passages [18,39,40]. While the traditional approach successfully models resistance emergence in environments with sub-inhibitory drug levels, it inherently excludes the evolutionary trajectories possible only under the bactericidal pressure. Our approach deliberately subjects bacterial populations to concentrations where only persisters or variants possessing exceptional immediate tolerance mechanisms can survive, simulating scenarios encountered at peak antibiotic concentrations or within poorly penetrated niches during aggressive chemotherapy. This high-stress environment imposes a dramatically different selective pressure compared to sub-MIC studies [41,42]. This design might provide novel insights into the resilience mechanisms and resistance evolution pathways.

Central to the evolved resistance observed under lethal meropenem stress were mutations in the well-characterized genes *mexR* and *oprD*. It has been demonstrated that mutations in the *mexR* and *oprD* genes result in upregulation of the multi-drug efflux system MexAB-OprM and a reduction in carbapenem uptake, respectively [43,44,45,46]. Crucially, our findings demonstrate that even under a bactericidal pressure far exceeding the MIC, these “classical” mechanisms retain substantial potency and were the dominant drivers of resistance development. This observation broadens the perceived applicability of these mutations. Prior studies extensively document their importance at sub-MIC or inhibitory concentrations in vitro and in vivo, where incremental increases in resistance enable survival and growth [47,48,49,50]. Our data show that the hyperexpression of the efflux system caused by *mexR* mutations and the impermeability caused by *oprD* loss are highly effective survival mechanisms under lethal dosages of meropenem. The persistent dominance of these mutations under lethal selective regimes underscores their important roles in *P. aeruginosa* carbapenem resistance and evidences their emergence during aggressive high-dose therapy.

In our passaging assays, *lasR*-null mutants emerged in the control groups, which is consistent with previous reports. In addition, *lasR* mutants have been identified from patients with chronic *P. aeruginosa* infections [51,52,53]. Clinically, *lasR* defects confer multidrug resistance (MDR) via efflux pump hyperactivation (e.g., MexXY/OprM- and NalC-mediated pathways), reducing intracellular antibiotic accumulation of β-lactams and aminoglycosides [54,55,56]. Inactivation of *lasR* correlates with mutations in efflux regulators (*nalC*, *mexZ*) [55,56]. However, *lasR* loss also imposes fitness costs. Mutants exhibit metabolic reprogramming toward anaerobic respiration via nitrate reductase (*nar*), enhancing survival in hypoxic biofilm niches but increasing susceptibility to nitrosative stress [57,58]. Crucially, this metabolic vulnerability may drive reversion to wild-type *lasR* under conditions where redox balance is critical [59,60]. Additionally, *lasR* mutants display bimodal persistence strategies: they enrich phenazine biosynthesis and EPS production, shielding persisters from immune clearance but compromise quorum-sensing (QS)-coordinated virulence, potentially reducing acute infectivity [58]. The epistatic interaction between *lasR* and secondary mutations further modulates resistance. As reported, *fusA1* mutations enhance MexXY efflux activity, amplifying tobramycin resistance in *lasR* mutants [59,60]. The absence of the *lasR* mutation in the meropenum treatment groups indicate that there might be a fitness cost of the *lasR* mutations under the pressure of a lethal dosage of meropenum. In vivo experiments are warranted to examine the development of *lasR* mutations in the presence of antibiotic treatment.

Beyond the established *mexR*/*oprD*-related mechanisms, genomic sequencing revealed mutations in genes not previously known to directly relate to meropenem resistance in *P. aeruginosa*. It has been reported that mutation of *sltB1* impairs the incorporation of a new peptidoglycan subunit into the cell wall, resembling low-level beta-lactam treatment, which leads to *ampC* upregulation and subsequent increased resistance to β-lactam antibiotics [61,62,63,64]. While the other genes lack established roles in drug efflux, enzymatic modification of antibiotics, or target alteration, the occurrence of their mutations in the resistant mutants suggests a potentially contributory role. We postulate that these mutations might increase bacterial tolerance, acting as crucial steppingstones under a lethal dosage of meropenem [65,66]. The mechanisms might be an alteration in metabolic flux, membrane composition, stress response pathways (e.g., envelope stress), or persister formation [67,68]. These early “enabler” mutations likely expand the population’s evolutionary opportunities by increasing bacterial survival under the lethal antibiotic stress [69]. This highlights a complex trajectory where initial stress adaptations preceded and potentiate the development of major resistance mutations under severe drug pressure. Functions of the early-stage mutated genes warrants further studies. A previous study in *Escherichia coli* demonstrated that resistant mutant cells in the bacterial population secrete indole, thereby improving the survival of the whole population [70]. Thus, the appearance of meropenem-resistant mutants might enhance the survival of the population, providing a bigger reservoir for additional mutations.

In addition, our study reveals that the meropenem-resistant mutants developed collateral resistance to ciprofloxacin and tobramycin. This phenomenon is most likely due to the *mexR* mutation, which results in upregulation of the MexAB-OprM efflux pump [43,44,45,46]. The collateral resistance may impair the treatment efficacies of ciprofloxacin and tobramycin despite no prior exposure [71]. Meanwhile, our data aligns with genomic surveys of XDR *P. aeruginosa* outbreaks, wherein *mexR* mutants exhibit 89% co-resistance to carbapenems, fluoroquinolones, and aminoglycosides, which confirmed this evolutionary trajectory’s relevance to clinics [72,73].

The passages in this study were performed in a complete LB medium in vitro. However, in clinical settings, bacteria encounter antibiotics in the host environment. The nutritional condition is significantly different from the in vitro culture medium. Meanwhile, the bacteria are under the pressure of the host’s immune clearance. This environment may alter the bacterial evolutionary trajectory of antibiotic resistance. For instance, mutations that increase antibiotic resistance while incurring fitness costs in the host environment are likely to be lost in vivo. Therefore, passaging assays in mouse infection models may reveal more clinic relevant bacterial evolutionary trajectories of antibiotic resistance.

## 5. Conclusions

This study delineates a hierarchical evolutionary trajectory in *P. aeruginosa* persister populations under lethal meropenem stress, which is different from conventional sub-inhibitory resistance evolution. We demonstrate that *mexR* and *oprD* mutations remain the dominant drivers of high-level meropenem resistance even under bactericidal pressure, confirming their major roles in efflux hyperactivation and porin-mediated impermeability against meropenem. Crucially, early mutations in *engA* and *sltB1*, previously unreported in carbapenem resistance, might function as evolutionary enablers, probably enhancing transient population survival and expanding the genetic reservoir for subsequent high-impact mutations. This adaptive cascade (enabler→*oprD* loss→*mexR*-mutation-mediated hyper-resistance) reveals an evolution trajectory under the lethal selective environments, providing a clue for understanding recalcitrant infections during high-dose therapy.

## Figures and Tables

**Table 1:** The MIC of single colonies on the 16 μg/mL meropenem plates at the 15th passage.

Strain	MIC (μg/mL)								
	MEM			CIP			TOB		
PA14	0.5			0.125			0.5		
Colony	1	2	3	1	2	3	1	2	3
Parallel-1	64	64	64	0.5	0.5	0.5	0.5	0.5	0.5
Parallel-2	64	64	64	0.5	0.5	0.5	0.5	0.5	0.5
Parallel-3	64	64	64	0.5	0.5	0.5	0.5	0.5	0.5

Data represent results from three independent experiments.

**Table 2:** The *mexR* and *oprD* mutations of the single colonies on the 16 μg/mL meropenem plates at the 15th passage.

Strain	*oprD* Mutation			*mexR*_T130P_ Mutation *		
Colony	1	2	3	1	2	3
Parallel-1	100D	37P	341D	+	+	+
Parallel-2	37P	37P	341D	+	+	+
Parallel-3	341D	899P	413P	+	+	+

*: “+” indicates the presence of *mexR*_T130P_ mutations in the colony.

**Table 3:** Bacterial antibiotic resistance levels (MIC) of *P. aeruginosa* strains.

Strain	Description	MIC (μg/mL)		
		MEM	CIP	TOB
**PA14**	Wild-type reference strain	0.5	0.125	0.5
**Δ** ***mexR***	*mexR* knockout	2	0.5	0.5
**Δ** ***mexR*** **/*mexR***	*mexR* knockout with pUC18T-mini-Tn7T-*mexR*	0.5	0.125	0.5
***mexR*** **_T130P_**	*mexR* mutation at the 130th amino acid: threonine to proline	2	0.5	0.5
***mexR*** **_T130P_** **/*mexR***	*mexR*_T130P_ with pUC18T-mini-Tn7T-*mexR*	0.5	0.125	0.5
**Δ*oprD***	*oprD* knockout	8	0.25	0.5
**Δ*oprD/*** **pUCP24-*oprD***	*oprD* knockout with pUCP24-*oprD*	0.5	0.125	0.5
**Δ*mexRΔoprD***	*mexR* knockout and *oprD* knockout	64	0.5	0.5
***mexR*** **_T130P_** ***ΔoprD***	*oprD* knockout and *mexR* mutation at the 130th amino acid: threonine to proline	64	0.5	0.5
**Δ*mexR*Δ*oprD*/** **pUCP24-*mexR*+*oprD***	*mexR* knockout and *oprD* knockout with pUCP24- *mexR*+*oprD*	1	0.125	0.5
***mexR*_T130P_Δ*oprD*/** **pUCP24-*mexR*+*oprD***	*oprD* knockout and *mexR* mutation at the 130th amino acid: threonine to proline with pUCP24-*mexR*+*oprD*	1	0.125	0.5

Data represent results from three independent experiments.

**Table 4:** Bacterial antibiotic resistance levels (MIC) of single colonies isolated from plates containing indicated concentrations of meropenem.

MEM Concentration	Passages	MIC (μg/mL)																	
		MEM						CIP						TOB					
	Colony	1	2	3	4	5	6	1	2	3	4	5	6	1	2	3	4	5	6
16 μg/mL	Parallel-1–11th	64	64	64	64	64	64	0.5	0.25	0.5	0.25	0.5	0.5	0.5	0.5	0.5	0.5	0.5	0.5
	Parallel-2–13th	64	64	64	64	64	64	0.5	0.5	0.25	0.5	0.5	0.5	0.5	0.5	0.5	0.5	0.5	0.5
	Parallel-3–14th	64	64	64	64	64	64	0.5	0.25	0.25	0.25	0.5	0.5	0.5	0.5	0.5	0.5	0.5	0.5
8 μg/mL	Parallel-1–10th	32	32	32	32	32	32	0.5	0.5	0.5	0.5	0.5	0.5	0.5	0.5	0.5	0.5	0.5	0.5
	Parallel-2–10th	32	32	32	32	32	16	0.5	0.5	0.25	0.5	0.5	0.5	0.5	0.5	0.5	0.5	0.5	0.5
	Parallel-3–11th	32	32	32	32	16	16	0.5	0.5	0.25	0.5	0.5	0.5	0.5	0.5	0.5	0.5	0.5	0.5
4 μg/mL	Parallel-1–8th	16	16	16	16	8	8	0.125	0.125	0.25	0.25	0.25	0.25	0.5	0.5	0.5	0.5	0.5	0.5
	Parallel-2–8th	16	16	8	8	8	8	0.5	0.5	0.25	0.25	0.5	0.5	0.5	0.5	0.5	0.5	0.5	0.5
	Parallel-3–8th	16	16	16	8	8	8	0.125	0.125	0.25	0.25	0.25	0.5	0.5	0.5	0.5	0.5	0.5	0.5
2 μg/mL	Parallel-1–7th	8	8	8	4	4	4	1	1	0.5	0.5	0.5	0.5	0.5	0.5	0.5	0.5	0.5	0.5
	Parallel-2–6th	8	4	4	4	4	4	0.125	0.125	0.125	0.125	0.125	0.125	0.5	0.5	0.5	0.5	0.5	0.5
	Parallel-3–6th	8	8	4	4	4	4	0.25	0.125	0.125	0.125	0.125	0.125	0.5	0.5	0.5	0.5	0.5	0.5

Data represent results from three independent experiments.

**Table 5:** Mutations in the *oprD* and *mexR* genes in stains isolated from plates containing indicated concentrations of meropenem.

MEM Concentration	Passages	*oprD* Mutations *						*mexR*_T130P_ Mutations *					
		1	2	3	4	5	6	1	2	3	4	5	6
16 μg/mL	11th-MEM-1	341D	341D, 734D	591D	105D	1183P	899P	+	+	+	+	+	+
	13th-MEM-2	341D	341D	341D	656P	710P	105D	+	+	+	+	+	+
	13th-MEM-3	341D	341D	341D	899P	899P	323D	+	+	+	+	+	+
8 μg/mL	10th-MEM-1	341D, 1204I	341D	760P	105D	591D	1024P	+	+	+	+	+	+
	10th-MEM-2	341D	341D	341D	805P	899P	105D	+	+	+	+	+	+
	11th-MEM-3	341D	341D	591D	105D	1183P	899P	+	+	+	+	+	+
4 μg/mL	8th-MEM-1	341D, 150P	341D	341D	793I, 805I	793I, 805I	899P						
	8th-MEM-2	341D	341D	341D	793I								
	8th-MEM-3	341D	341D	954P	793I								

*: D, P, and I represent the occurrence of deletion mutation, point mutation, and insertion mutation, respectively, at the corresponding positions. “+” indicates the presence of *mexR*_T130P_ mutations in the colony, and a space indicates the absence.

## Data Availability

The original data presented in the study are openly available in the NCBI SRA database at accession number PRJNA1268141.

## Supplementary Materials

The following supporting information can be downloaded at: https://www.mdpi.com/article/10.3390/microorganisms13071672/s1, Table S1: Bacteria and plasmids used in this study; Table S2: Primers used in this study; Table S3: Group 1 7th-generation mutation genes; Table S4: Group 2 6th-generation mutation genes; Table S5: Group 3 6th-generation mutation genes. Figure S1: Group 1 generation 7th allele mutant frequency trajectories; Figure S2: Group 2 generation 6th allele mutant frequency trajectories; Figure S3: Group 3 generation 6th allele mutant frequency trajectories. References [74,75] are cited in the Supplementary Materials.

## Abbreviations

The following abbreviations are used in this manuscript:

MEM	meropenem
CIP	ciprofloxacin
TOB	tobramycin
