# Supplementary material for: Evolution of Resistant Mutants in Pseudomonas aeruginosa Persister Cells Under Meropenem Treatment
Source: Microorganisms. 2025 Jul 16;13(7):1672. doi: 10.3390/microorganisms13071672 (PMC12299539; doi:10.3390/microorganisms13071672)
Supplement: Supplementary file 1 [file microorganisms-13-01672-s001.zip › microorganisms-3704062-supplementary.pdf]

Table S1 Bacteria and plasmids used in this study.

Bacteria	Description	Source
PA14	*mexR* knockout	Lab stock
PA14Δ*mexR*	*mexR* knockout with pUC18T-mini-Tn7T-*mexR*	This study
PA14Δ*mexR*/*mexR*	*mexR* mutation at the 130th amino acid: threonine to proline	This study
PA14$mexR_{T130P}$	$mexR_{T130P}$ with pUC18T-mini-Tn7T-*mexR*	This study
PA14$mexR_{T130P}$/*mexR*	*mexR* knockout	This study
PA14Δ*oprD*	*oprD* knockout with pUCP24-*oprD*	This study
PA14Δ*oprD*/pUCP24-*oprD*	*mexR* knockout and *oprD* knockout	This study
Plasmids		
pEX18Tc	Gene replacement vector; TET^r, $oriT^+$, $sacB^+$	[1]
pEX18Tc-Δ*mexR*	*mexR* gene deletion on pEX18Tc	This study
pUC18T-mini-Tn7T-Gm	Mini-Tn7 base vector from insertion into chromosome attTn7 site; GEN^r	[1]
pTNS3	Helper plasmid, for gene insertion in chromosome; AMP^r	[1]
pUC18T-mini-Tn7T-Gm-*mexR*	pUC18T-mini-Tn7T-Gm with *mexR*, GM^r	This study
pUC18T-mini-Tn7T-Gm- $mexR_{T130P}$	pUC18T-mini-Tn7T-Gm with $mexR_{T130P}$, GM^r	This study
pEX18Tc-Δ*oprD*	*oprD* gene deletion on pEX18Tc	This study
pUCP24	Expression vector with *tac* promoter; Gm^r	[2]
pUCP24-*oprD*	pUCP24 with *oprD*, GM^r	

[1] Choi KH, Schweizer HP. mini-Tn7 insertion in bacteria with single attTn7 sites: example Pseudomonas aeruginosa. Nat Protoc. 2006;1(1):153-61. doi: 10.1038/nprot.2006.24. PMID: 17406227.

[2] Heurlier K, Williams F, Heeb S, Dormond C, Pessi G, Singer D, Cámara M, Williams P, Haas D. Positive control of swarming, rhamnolipid synthesis, and lipase production by the posttranscriptional RsmA/RsmZ system in Pseudomonas aeruginosa PAO1. J Bacteriol. 2004 May;186(10):2936-45. doi: 10.1128/JB.186.10.2936-2945.2004. PMID: 15126453; PMCID: PMC400603.

Table S2 Primers used in this study.

Primer	Sequence 5'-3'	Function	Source
mexR-up-F	CGGAATTCTGAGGATGATGCCGTTCA	For deletion of *mexR*	This study
mexR-up-R	G GAGCTCTGGTTTGGCCGAGTAAAC		This study
mexR-dw-F	GGAGCTCGAACATTCTTTTCGAAGC		This study
mexR-dw-R	CCAAGCTTCTGTACGGCCGCTTCAAC		This study
mexR-com-F	CCAAGCTTTGAGGATGATGCCGTTCA	For complementation of *mexR*	This study
mexR-com-R	GGGGTACCTTACATCATCACCATCACCAT AATATCCTCAAGCGG		This study
mexR-T130P-F	CGGAATTCTGAGGATGATGCCGTTCA	For *mexR* T130P mutation	This study
mexR-T130P-R	GCTCTAGACTGTACGGCCGCTTCAAC		This study
mexR(A)-F	TCACCCCGGAGGAACAAGCCA	For test *mexR* T130P mutation	
mexR(C)-F	TCACCCCGGAGGAACAAGCCC		
mexR-R	CTGTACGGCCGCTTCAACGACTTC		
oprD -up-F	CGAGCTCGATCACGTGCATGTGGAG	For deletion of *mexR*	This study
oprD -up-R	TTGCCTGTCGGTCGATGTGATTGCTCCTTT		This study
oprD -dw-F	AAAGGAGCAATCACATCGACCGACAGGCAA		This study
oprD -dw-R	CCAAGCTTTCGCGGAACTTGGTCTGG		This study
oprD-com-F	TATGACCATGATTACGAATTCAATAATTTCAAAAC CAAAGG	For complementation of *oprD*	This study
oprD-com-R	ACGACGGCCAGTGCCAAGCTTTTACAGGATCGAC AGCGGAT		This study

Table S3 Group 1 generation 7th mutation genes.

PA14 ID	PAO1 ID	Description	POS*	REF*	ALT*	DEPTH	Allele Frequency	In group 1 generation 15th Allele Frequency
**PA14_01160**	**PA0095**	*vgrG1b*	114794	A	G	738	0.2818	0.2135
			114865	T	C	708	0.1793	0.1560
PA14_01580	PA0129 bauD Amino acid permease	*gabP* gamma-aminobutyrate permease	144240	T	G	470	0.0617	0.1415
PA14_09460	PA4212	*phzC1*	810913	G	A	429	0.0559	-
PA14_12080	PA4001	*sltB1* soluble lytic transglycosylase B	1044755	A	C	424	0.0542	-
PA14_14680	PA3818	extragenic suppressor protein SuhB	1251670	G	T	300	0.0633	-
PA14_14930	PA3799	GTP-binding protein EngA	1268137	A	C	261	0.0957	-
PA14_20260	PA3388		1745821	C	A	329	0.0516	-
PA14_23370		*orfK* UDP-N-acetylglucosamine 2-epimerase	2028376	C	T	474	0.1118	-
PA14_30970			2691468	A	G	438	0.0730	-
PA14_32190	antR		2798927	A	C	185	0.1135	-
PA14_34490	PA2330	oxidoreductase activity	3065486	C	A	80	0.0625	-
PA14_36710	PA2153	*glgB* glycogen branching protein	3271409	C	A	196	0.0510	-
**PA14_39925**	**PA1902**	*phzD2*	3559401	T	C	60	0.9666	1
PA14_43080	PA5266 vgrG6	*vgrG14*	3832626	C	T	319	0.1285	0.1418
PA14_50670	PA1060		4501312	T	G	285	0.0596	-
PA14_51650		integrase	4590213	T	C	548	0.1551	0.0837
PA14_55320	PA0696		4911840	A	C	305	0.0557	-
PA14_58130	PA4480	rod shape-determining protein MreC	5174987	G	T	145	0.0551	-
PA14_63620	PA4813	lipase LipC	5673071	T	G	418	0.0933	-
PA14_63940	PA4836	*cntL*	5696000	T	G	475	0.0968	-
**PA14_61200**	**PA4625**	cyclic diguanylate-regulated TPS partner A, CdrA	5460217	A	G	522	0.0804	0.1156
			5460283	A	G	557	0.1615	0.1234
			5460364	A	G	502	0.0637	0.0714
			5461009	A	G	623	0.2070	0.2796
			5461039	A	G	696	0.2773	0.4510
			5461135	A	G	815	0.4736	-
			5461777	G	C	561	0.1301	-

*POS: The genomic position of the mutation site. REF: The reference sequence at the position. ALT:The mutated sequence at the position.

Table S4 Group 2 generation 6th mutation genes.

PA14 ID	PAO1 ID	Description	POS*	REF*	ALT*	DEPTH	Allele Frequency	In group 1 generation 15th Allele Frequency
**PA14_01160**	**PA0095**	*vgrG1b*	114794	A	G	849	0.2897	0.1761
			114865	T	C	752	0.2181	0.1108
PA14_23400			2032400	A	C	535	0.2673	-
**PA14_39925**	**PA1902**	*phzD2*	3559401	T	C	73	1	0.9778
PA14_43080	PA5266 vgrG6	*vgrG14*	3832569	T	A	406	0.1724	0.1436
			3832626	C	T	407	0.1548	0.1224
PA14_47880	PA1264	transcriptional regulator	4261290	T	C	290	0.1483	-
PA14_51650		integrase	4590213	T	C	668	0.1003	0.0414
**PA14_61200**	**PA4625**	cyclic diguanylate-regulated TPS partner A, CdrA	5461009	A	G	789	0.2193	0.2386
			5461039	A	G	886	0.2810	0.2532
			5461135	A	G	1036	0.4759	0.4970
			5461777	G	C	657	0.1096	-

*POS: The genomic position of the mutation site. REF: The reference sequence at the position. ALT:The mutated sequence at the position.

Table S5 Group 3 generation 6th mutation genes.

PA14 ID	PAO1 ID	Description	POS*	REF*	ALT*	DEPTH	Allele Frequency	In group 1 generation 15th Allele Frequency
**PA14_01160**	**PA0095**	*vgrG1b*	114794	A	G	1113	0.3126	0.2040
			114865	T	C	970	0.2433	0.1285
PA14_01580	PA0129 bauD	*gabP*	144240	T	G	627	0.0590	-
PA14_10360	PA4141		895338	A	C	457	0.0656	0.1395
			895378	A	C	496	0.0585	-
PA14_23400			2032967	C	T	652	0.1810	-
PA14_25500	PA2982		2229431	A	C	534	0.0599	-
PA14_27050	PA2865	probable glycosylase	2351391	C	T	288	0.0625	-
PA14_27890	PA2803		2415011	G	T	345	0.0550	-
PA14_28980	PA3900	*fecR*	2505541	G	T	363	0.0716	-
PA14_33280	PA2424	*pvdL*	2924166	T	G	338	0.0740	-
PA14_34820	PA2304 ambC		3095081	A	C	229	0.0524	-
PA14_38500	PA2010	*hmgR*	3435037	A	C	254	0.0906	-
**PA14_39925**	**PA1902**	*phzD2*	3559401	T	C	70	1	1
PA14_41350	PA1796	*folD* 5,10-methylene-tetrahydrofolate dehydrogenase /cyclohydrolase	3688211	A	C	340	0.0559	-
PA14_43080	PA5266 vgrG6	*vgrG14*	3832626	C	T	454	0.0947	-
PA14_44740	PA1523	*xdhB* xanthine dehydrogenase	3987321	T	G	352	0.0625	-
PA14_47880	PA1264	probable transcriptional regulator	4261290	T	C	301	0.1063	-
PA14_53900	PA0799	probable helicase	4779548	T	G	366	0.1038	-
**PA14_61200**	**PA4625**	cyclic diguanylate-regulated TPS partner A, CdrA	5460217	A	G	641	0.0764	0.0827
			5460283	A	G	707	0.1556	0.0891
			5460364	A	G	665	0.0586	0.0809
			5461009	A	G	814	0.1916	0.2738
			5461039	A	G	898	0.2595	0.2938
			5461135	A	G	1054	0.4886	0.4854
			5461777	G	C	698	0.1017	-
PA14_68360	PA5174	beta-acetoacetyl-acyl carrier protein synthase FabY	6098904	T	G	630	0.0508	-

*POS: The genomic position of the mutation site. REF: The reference sequence at the position. ALT:The mutated sequence at the position.

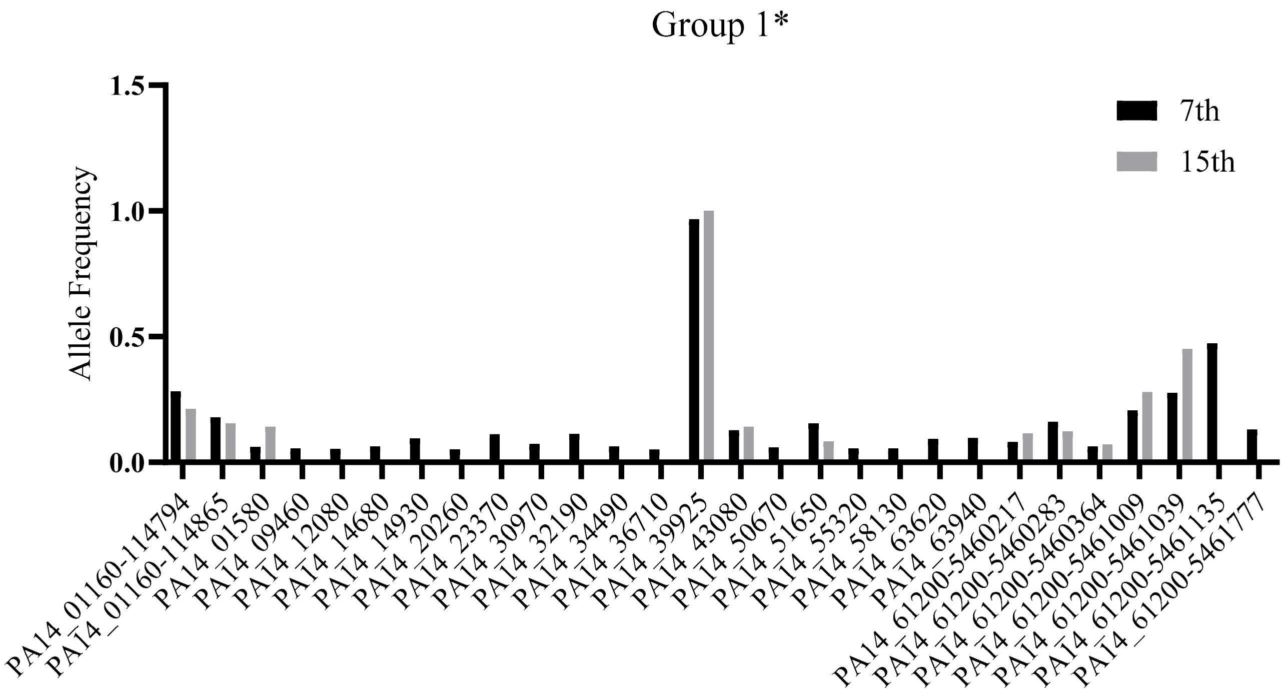

Figure S1 Group 1 generation 7th allele mutant frequency trajectories.

* Genes are annotated using the gene IDs of PA14. For genes with multiple mutation sites, the genomic positions of the mutation sites are appended to their respective gene IDs.

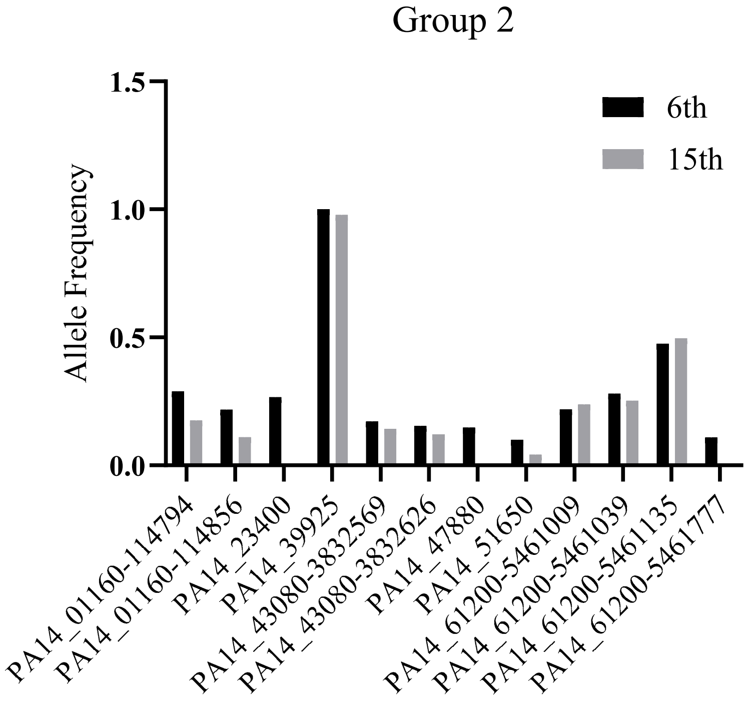

Figure S2 Group 2 generation 6th allele mutant frequency trajectories.

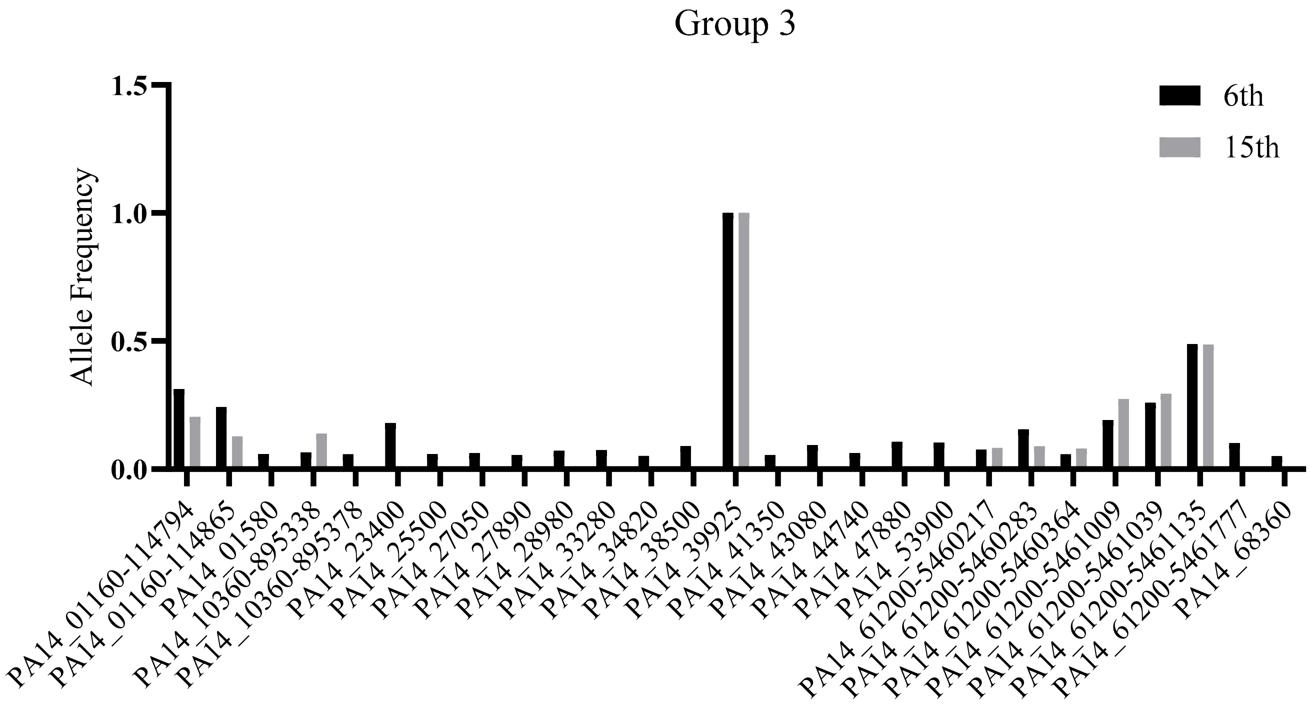

Figure S3 Group 3 generation 6th allele mutant frequency trajectories.
